# Insights Into Human Intrahepatic NK Cell Function From Single Cell RNA Sequencing Datasets

**DOI:** 10.3389/fimmu.2021.649311

**Published:** 2021-03-22

**Authors:** Gráinne Jameson, Mark W. Robinson

**Affiliations:** ^1^School of Medicine, Trinity Translational Medicine Institute, Trinity College Dublin, Dublin, Ireland; ^2^Department of Biology, Kathleen Lonsdale Institute for Human Health Research, Maynooth University, Maynooth, Ireland

**Keywords:** liver, RNA-seq, liver-resident, intrahepatic, NK cell

## Abstract

Diverse populations of natural killer (NK) cells have been identified in circulating peripheral blood and a wide variety of different tissues and organs. These tissue-resident NK cell populations are phenotypically distinct from circulating NK cells, however, functional descriptions of their roles within tissues are lacking. Recent advances in single cell RNA sequencing (scRNA-seq) have enabled detailed transcriptional profiling of tissues at the level of single cells and provide the opportunity to explore NK cell diversity within tissues. This review explores potential novel functions of human liver-resident (lr)NK cells identified in human liver scRNA-seq studies. By comparing these datasets we identified up-regulated and down-regulated genes associated with lrNK cells clusters. These genes encode a number of activating and inhibiting receptors, as well as signal transduction molecules, which highlight potential unique pathways that lrNK cells utilize to respond to stimuli within the human liver. This unique receptor repertoire of lrNK cells may confer the ability to regulate a number of immune cell populations, such as circulating monocytes and T cells, while avoiding activation by liver hepatocytes and Kupffer cells. Validating the expression of these receptors on lrNK cells and the proposed cellular interactions within the human liver will expand our understanding of the liver-specific homeostatic roles of this tissue-resident immune cell population.

## Introduction

### Natural Killer Cells: From Blood to Tissues

The phenotype and function of natural killer (NK) cells have been extensively studied in both mice and humans, predominantly utilizing NK cells isolated from circulating peripheral blood (PB). Circulating NK cells account for 5–15% of the total lymphoid population in PB. In humans two functionally distinct subsets of NK cells are recognized, based on their expression of CD56 and CD16: CD56^dim^CD16^+^ and CD56^bright^CD16^lo/−^ (1). The CD56^dim^ NK cells represent the majority of circulating NK cells (~90%) and are more cytotoxic than CD56^bright^ NK cells (2).

Circulating NK cells are equipped to recognize and kill both tumor and virally infected cells as well as possessing the ability to regulate other immune cells (3, 4). To carry out their effector functions NK cells have an extensive repertoire of stimulatory, costimulatory and inhibitory receptors but, unlike T and B cells, the genes encoding these receptors do not undergo somatic gene recombination. These NK cell receptors include the killer immunoglobulin-like receptor (KIR) family, the CD94(KLRD1)/NKG2 family of C-type lectins, the natural cytotoxicity receptors (NCR) and as well as a variety other activating and inhibitory surface receptors, as reviewed previously (5–7). It is the balance of expression of these receptors that is necessary to discriminate between healthy host cells and cells in distress, as well as self and non-self-cells. This balance ensures NK cells remain tolerant of healthy tissue whilst effectively clearing any potential threats, such as viral infection or cancerous cells.

In addition to these circulating NK cell populations it is evident that a variety of tissue-resident NK cell subsets exist (8). Tissue-resident NK cells are enriched in lymphoid tissues, the uterus and the liver, as well as a number of other tissues (9–12). Tissue-resident NK cell populations are predominantly CD56^bright^ in humans and they appear to be phenotypically and functionally distinct from their circulating counterparts (8). In the human liver NK cells constitute up to half of total lymphocytes. The majority of intrahepatic NK cells possess a CD56^bright^ phenotype, however their functional roles within the liver remains unclear (8, 13–16).

## The Liver as an Immunological Organ

The liver has vital metabolic functions, detoxification functions, and immunological functions (17, 18). It is home to a diverse repertoire of resident immune cells including Kupffer cells (KC), NK cells and other innate and adaptive immune cells which are thought to contribute to maintain hepatic tolerance (19–21). The liver receives up to 80% of its blood supply from the gut via the hepatic portal vein, and this blood contains a high concentration of foreign molecules derived from our diet and gut microbiome. Resident immune cells must remain tolerant to this continuous exposure to harmless dietary and commensal bacterial products, while simultaneously being ready to clear any pathogens, malignant cells or toxic products that it may encounter, ensuring organ homeostasis (18).

### Intrahepatic NK Cells

Intrahepatic NK cells were first described in rat livers as “pit cells” in the 1970s after their cytoplasmic granules which resembled grape pits (22). For many years it was known that NK cells were enriched in the liver, however the functional significance of this was unknown (14, 23). Murine studies investigating memory-like NK cell responses identified a population of intrahepatic NK cells in murine livers defined as DX5^−^CD49a^+^CXCR6^+^TRAIL^+^ (24– 28). In 2015 an equivalent human intrahepatic NK cell population was described within hepatic sinusoids defined by the expression of CD49a (29). These CD49a^+^ intrahepatic NK cells display enhanced pro-inflammatory cytokine responses and memory-like recall responses in *in vivo* murine and *ex vivo* human studies (25, 29, 30).

In human liver this CD49a^+^ intrahepatic NK cell population is highly variable between donors and only represents a minority of the total intrahepatic NK cell population. The majority of human intrahepatic NK cells consist of a CD56^bright^ liver-resident (lr)NK cell population, and are defined phenotypically as CD56^bright^CXCR6^+^CD69^+^Eomes^hi^Tbet^lo^CD49a^−^CD49e^−^ (11, 13, 16, 29, 31–33). Due to the species specific differences between mice and humans this review will focus only on human studies, with the term “lrNK” being used to specifically refer to the human CD56^bright^CXCR6^+^CD69^+^Eomes^hi^Tbet^lo^CD49a^−^CD49e^−^ NK cell population.

Using these defined phenotypical markers the effector function of human lrNK cells have been explored in a number of studies (summarized in Table 1). These studies have identified equivalent or reduced cytokine responses and increased degranulation compared PB NK cells and intrahepatic CD56^dim^ populations (Table 1). However, to date these findings are limited to functional assays assessing a limited range of markers, upon stimulation with cytokines or HLA class I deficient immortalized cell lines. In this context, data generated from single cell RNA sequencing (scRNA-seq) studies can provide novel insights into potential roles for human lrNK cells beyond IFN-γ and TNF-α production and cytotoxic responses to cytokine activation or HLA-deficient tumor cells.

**Table 1:** Overview of studies that have investigated human lrNK cell function.

**References**	**Intrahepatic NK cell subset (A)**	**Comparison cell subset (B)**	**Functional difference (A) vs. (B)**	**Stimulation**
Harmon et al. (16)	Hepatic CD56^bright^	PB CD56^bright^	Enhanced CD107α and reduced IFN-γ expression	MHC Class I deficient K562 target cells effector:target 5:1 for 4 h either with or without overnight rhIL-2 priming
Hudspeth et al. (13)	Hepatic CD56^bright^	PB CD56^dim^	Similar IFN-γresponses	20 ng/ml rhIL-12 and 200 U/ml rhIL-2 for 18 h
Stegmann et al. (11)	Hepatic CXCR6^+^	Hepatic CXCR6^−^	Lower IFN-γresponses	IL-12/IL-18 (5 ng/ml, 50 ng/ml) for 4 h
Aw Yeang et al. (33)	Hepatic CD49e^−^	Hepatic CD49e^+^	Similar IFN-γ and TNF-αresponses	PMA (20 ng/ml) and ionomycin (1 mg/ml) for 6 h
Sun et al. (34)	Hepatic CD160^+^	Hepatic CD160^−^	Higher basal IFN-γ	Unstimulated
Lunemann et al. (35)	Hepatic CXCR6^+^CD56^bright^	Hepatic CXCR6^−^CD56^bright^	Reduced IFN-γ and TNF-α	0.221 cells at an effector:target cell ratio of 5:1 for a total of 6 h
Zhao et al. (36)	Hepatic CXCR6^+^CD16^−^	PB and hepatic CXCR6^−^CD16^+^	More CD107α vs. both PB and hepatic CXCR6^−^CD16^+^ More IFN-γ vs. hepatic CXCR6^−^CD16^+^	IL-12/IL-18 (50 ng/ml) for 6 h

## Single Cell RNA Sequencing Analysis of Intrahepatic Immune Cell Populations

Recent advancements in scRNA-seq have provided us with a detailed view of the cellular components that make up the human liver (17, 32, 36–39). A number of studies utilizing scRNA-seq technologies have profiled the human liver (summarized in Table 2) (36–40). These studies have provided a global overview of the unique liver-specific adaptations of both parenchymal and non-parenchymal cells, including tissue-resident immune cells. This data represents an important resource for the international research community.

**Table 2:** Summary of human liver scRNA-seq studies and the NK cell clusters identified.

**References**	**Year**	**Journal**	**Sample type**	**Technology platform**	**Cells sequenced**	**GEO dataset ID**	**NK cell clusters**	**Descriptor of gene sets available**
Zhao et al. (36)	2020	Cell Discovery	Liver perfusate obtained during orthotopic liver transplantation (*n* = 3); Fresh	Library = 10X Genomics, Sequenced = Illumina Hiseq XTEN platform	Magnetically sorted CD45^+^ cells	GSE125188	Clusters 12 and 13; Figure 2, Supplementary Table 4	DEG comparing CXCR6+ NK vs. CX3CR1+ NK cell clusters (up-regulated genes in each cluster)
Zhang et al. (40)	2020	Journal of Hepatology	Treatment naïve-ICC tissues and paired adjacent normal samples	Library = 10X Genomics, Sequenced = Illumina Xten or NovaSeq 6000 system	Viable single cell suspension post tissue digestion	GSE138709, GSE142784	NK cell; Supplementary Table 2	DEG comparing NK cell cluster vs. all other clusters (no information on up- or down-regulation)
Aizarani et al. (38)	2019	Nature	Healthy liver tissue obtained during resections for CRC metastasis or CC (*n* = 9); Cryopreserved and fresh	mCEL-Seq2	PHH and NPCs or sorted directly after tissue digestion if HCC sample.	GSE124395	Cluster 5; Supplementary Table 1	DEG comparing NK cell cluster vs. all other clusters (up- and down-regulated genes)
Ramachandran et al. (39)	2019	Nature	Healthy liver tissue obtained during resections for CRC metastasis (*n* = 5) Cirrhotic tissue obtained during orthotopic liver transplantation (*n* = 5); Fresh	Library = 10X Genomics/Sequenced= Illumina HiSeq 4000	Viable CD45^+^ leucocytes vs. CD45^−^ non-parenchymal cells were sorted via FACS	GSE136103	Clusters NK cell (1), NK cell (2), and cNK cell; Extended Data Figure 3E, Supplementary Table 6	DEG comparing each NK cell cluster vs. all other clusters (up-regulated genes in each cluster)
MacParland et al. (37)	2018	Nature Communictions	Healthy liver tissue obtained during orthotopic liver transplantation (*n* = 5); Fresh	Library = 10X Genomics/Sequenced= Illumina HiSeq 2500	Total liver homogenate	GSE115469	Cluster 8; Supplementary Data 1	DEG comparing NK cell cluster vs. all other clusters (up- and down-regulated genes)

*CRC, Colorectal cancer; CC, cholangiocarcinoma; PHH, primary human hepatocytes; NPCs, non-parenchymal cells; FACS, fluorescence-activated cell sorting; DEG, differentially-expressed genes*.

These scRNA-seq datasets have sampled all tissue-resident immune cell populations (limited only by the number of single cells sequenced in each dataset). However, subsequent validation experiments and functional work in these studies have, out of necessity, focused on only a few selected cell populations present in the human liver. One population currently lacking a detailed interpretation of the transcription profiles generated in these datasets is lrNK cells.

In a number of published scRNA-seq studies of the human liver NK cell clusters have been identified although the composition of these NK cell clusters varies between studies (Table 2). The requirement to define clusters based on genes characteristic of specific cell types represents a limitation of scRNA-seq studies. While phenotypic markers have been well defined, the transcriptional profiles that distinguish between immune cell subpopulations, in particular subpopulations of NK cells, lack extended validation. This issue is compounded by the fact that the optimal number of clusters differs depending on the specific research question. The datasets generated by MacParland et al. (37), Aizarani et al. (38), and Zhang et al. (40), all identified a single cluster of NK cells (Table 2). It is likely this single NK cell cluster contains both lrNK cells and circulating NK cells. As such, further analysis of NK cell subpopulations is not possible without reanalysing the raw sequencing datasets in order to generate additional NK cell clusters.

In contrast both the study by Zhao et al. (36) and the study by Ramachandran et al. (39) identified multiple NK cell clusters (Table 2). The study by Zhao and colleagues identified differentially expressed genes comparing between CXCR6^+^ lrNK cells and CX3CR1^+^ circulating (c)NK cells (36). The study by Ramachandran and colleagues identified two lrNK cell clusters and one cNK cell cluster, and included lists of differentially expressed genes comparing each individual cluster to all others (39). This study includes liver samples from both healthy controls and patients with liver cirrhosis, and one of the lrNK cell clusters is largely absent in cirrhotic patients highlighting the possibility that NK cell subpopulations may be altered in the diseased liver (39).

**Figure 1:**
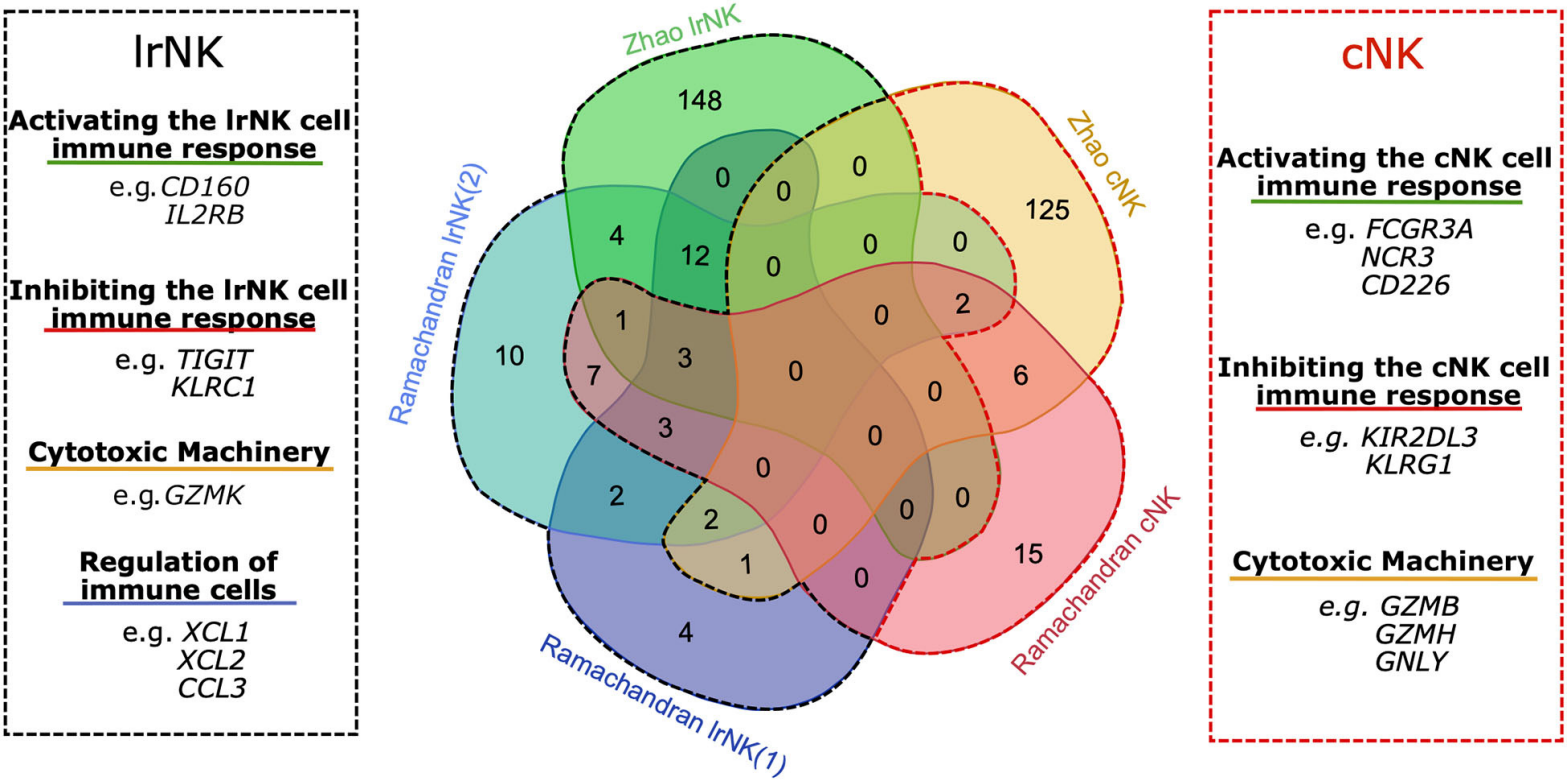
Overlap of up-regulated genes in lrNK cell populations from human liver scRNA-seq datasets. Venn diagram illustrating the overlap of DEG within each NK cell cluster of both the Zhao et al. (36) and Ramachandran et al. (39) datasets. The black dashed line and the red dashed line indicate genes up-regulated in lrNK cell clusters and cNK clusters respectively.

To explore the transcriptional profile unique to lrNK cells our review focuses on genes identified in the Ramachandran et al. and Zhao et al. datasets (36, 39). Comparing the lrNK cell and cNK cell clusters between these 2 datasets identifies 180 up-regulated genes associated with lrNK cells, 146 up-regulated genes associated with cNK cells and 19 up-regulated genes shared between lrNK and cNK clusters (Figure 1 and Supplementary Data). Genes up-regulated in lrNK cell clusters include *EOMES* and *CXCR6*, which have been validated at a protein level as phenotypic markers of lrNK cells (11, 13, 16). Genes up-regulated in cNK cell clusters include *ITGAM, S1PR1*, and *SELL*, which are markers of immune cell migration that are down-regulated at a protein level in tissue-resident immune cell populations (31, 33). A variety of activating and inhibitory receptors, downstream signaling molecules, and effector molecules are differentially expressed between lrNK cells and cNK cells (Figure 1 and Supplementary Data). These differentially regulated genes provide opportunities to identify novel markers of lrNK cells as well as providing intriguing insights into potential functions of lrNK cells within the human liver, which are distinct from cNK cells and extend beyond the recognition of virally infected cells or tumors.

### Activating the lrNK Cell Immune Response

A number of genes involved in the activation of NK cells are differentially regulated in lrNK and cNK cells in these scRNA-seq datasets (Figure 1 and Supplementary Data). The lrNK cell clusters up-regulate activating receptor genes *CD160, CD27, CD7, IL2RB, TMIGD2*, and *TNFSF14* as well as *SH2D1A*, which is involved in signal transduction. In contrast the cNK cell clusters up-regulate activating receptor genes such as *CD226, FCGR3A, IL12RB1*, and *NCR3*.

A number of these genes are involved in NK cell interactions with hepatocytes and other immune cell populations. *IL2RB* encodes the interleukin-2 receptor β-chain (IL-2Rβ; CD122) and is a major marker of NK-committed cells or NK-progenitors, which allows the cells to respond to IL-15 (41). IL-2Rα forms part of the IL-2 as well as the IL-15 receptor (in complex with IL-15Rα) (42). IL-15 is important for NK cell survival, activation and function (43, 44), is strongly expressed in the liver (45–47), and hepatocyte- and KC-derived IL-15/IL-15Rα directly regulates the homeostasis of liver NK cells via trans-presentation (48). Conversely *IL12RB*, which encodes IL-12Rβ, is upregulated on cNK cells and induces IFN-γ production when stimulated by IL-12, highlighting that cNK and lrNK cells are regulated by different cytokines in the liver microenvironment (49).

CD27^+^ NK cells are rare in the PB yet are enriched in tissues and associated with lower cytotoxic abilities with reduced granzyme B and perforin in comparison to their CD27^−^ counterparts (50). Interestingly, upon binding of its ligand CD70, CD27 is downregulated in an IL-15R-dependent manner suggesting that these lrNK cell's cytotoxicity is tightly controlled by their expression of IL-15R and CD27 and the local availability of IL-15 and CD70 (51). In the steady state, CD70 levels are quite low, with tightly regulated expression observed upon activation on T cells, B cells and subsets of DCs (52).

Both *CD160* and *TNFSF14* (also known as *LIGHT*) encode proteins capable of binding to herpes virus entry mediator (HVEM; also known as TNFRSF14) (53, 54). HVEM is widely expressed by monocytes, dendritic cells (DC), neutrophils, NK cells, T- and B-cells and downregulated in activated T cells (55, 56). CD160 also binds weakly to classical MHC-I such as HLA-C (57, 58) and the non-classical MHC-I soluble HLA-G (59), while LIGHT also binds to lymphotoxin-receptor (LT-R, also known as TNFRSF3) (60).

Engagement of CD160 or LIGHT on PB CD56^dim^ NK cells activates their cytotoxic effector function enhancing NK-mediated lysis of target cells (54, 58, 61), indicating the possibility that NK cells directly regulate HVEM-expressing immune cells in the liver. Both KCs and hepatocytes express LT-R within the liver. The engagement of LIGHT and LT-R on hepatocytes has also been shown to regulate hepatic lipase expression (62) as well as directly regulating hepatocyte proliferation and liver regeneration in response to partial hepatectomy (63–65).

*CD7* encodes an early differentiation marker on common lymphoid progenitors and crosslinking of CD7 induces NK cell activation, proliferation, cytokine production and cytotoxicity (66, 67). Ligands for CD7 include SECTM1 (68) (secreted and transmembrane protein 1) and LGALS1 (galectin-1) (69). SECTM1 is highly expressed by epithelial cells and acts as a chemoattractant for CD7 expressing cells (70, 71). Galectin-1 is also expressed by epithelial cells as well as T regulatory cells. Galectin-1 is capable of suppressing inflammatory immune responses and is essential for regulating inflammation and hepatocyte proliferation during liver regeneration (72).

*SH2D1A* encodes the signaling lymphocyte activation molecule (SLAM)-associated protein SAP. This adaptor protein mediates downstream signaling from SLAM family receptors on NK cells leading to stable conjugate formation with target cells via LFA-1 (73) and target cell-directed cytotoxic granule polarization and exocytosis (74). SLAM family receptors are immunoglobulins expressed on a variety of immune cell populations that are activated via homophilic binding (except for SLAMF4/2B4 which binds SLAMF2/CD48) (75).

In addition to these up-regulated genes, a number of activating receptors are expressed by cNK cell but not lrNK cells. cNK cells upregulate *FCGR3A* which encodes the CD16 protein, a key marker of CD56^dim^ cNK cells, which is responsible for antibody-dependent cell-mediated cytotoxicity (ADCC). *NCR3* encodes NKp30 and is upregulated on cNK cells equipping them with anti-tumor specific cytotoxic abilities (76). Likewise the *CD226* gene is upregulated on cNK cells and encodes the activating receptor DNAM-I. Interestingly this receptor shares ligands, which include polio virus receptor (PVR/CD155) and Nectin-2 (PVRL2/ CD112), with the inhibitory molecule T cell Ig and ITIM domain (TIGIT) that is upregulated in lrNK cells and is discussed in detail in the next section (77). This differential expression emphasizes how certain stimuli can regulate cNK and lrNK cell populations in different directions.

**Figure 2:**
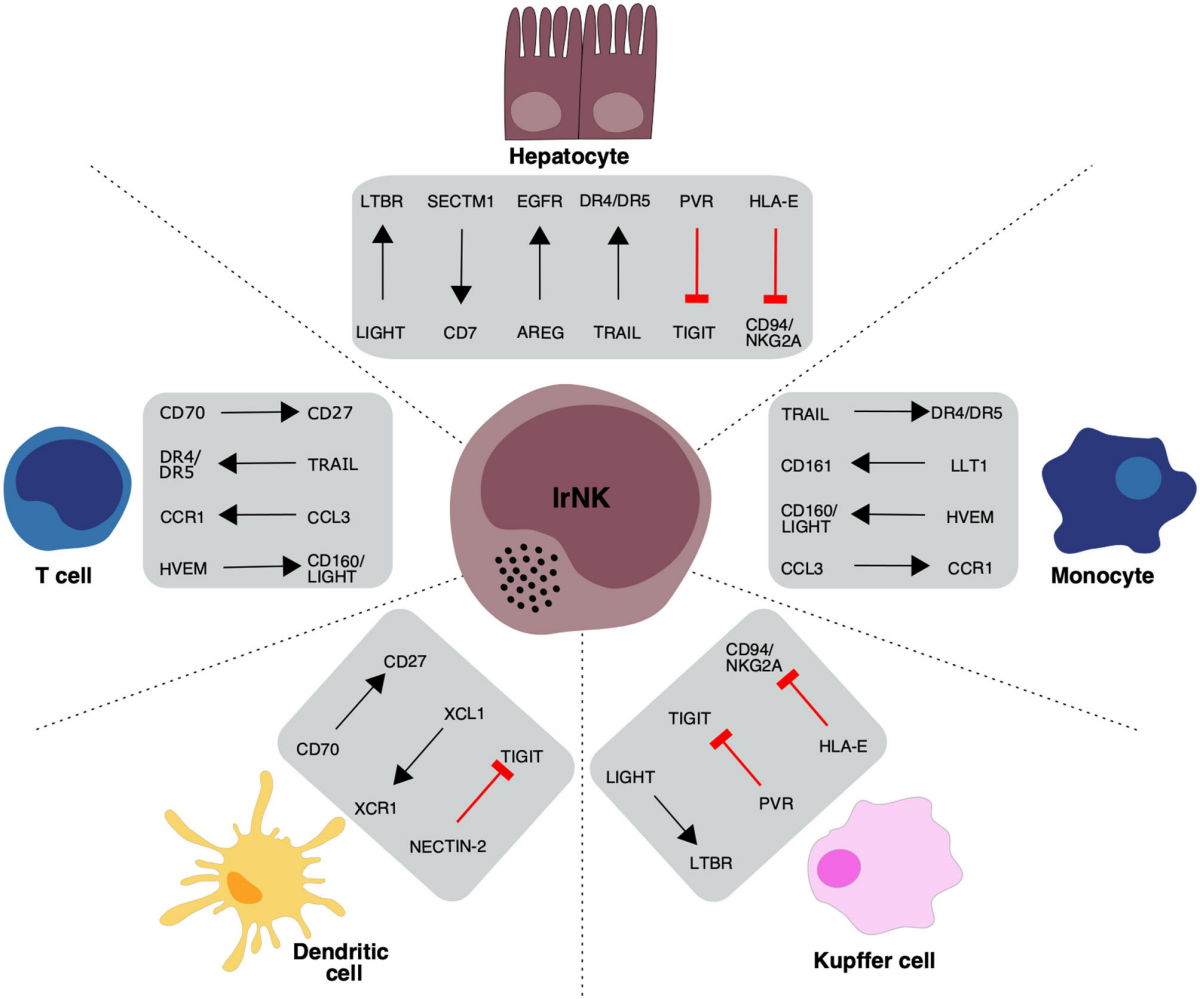
Overview of proposed receptor-ligand interactions between lrNK cells and haemopoietic and non-haemopoietic cell populations in the liver. Black arrows indicate the direction of a stimulatory ligand-receptor interaction and red lines indicate an inhibitory ligand-receptor interaction between interacting cell types.

These differentially expressed activating receptor genes highlight the importance of hepatocytes in regulating lrNK cell survival and imply an important role for lrNK cells in regulating infiltrating immune cell populations within the human liver (Figure 2).

### Inhibiting the lrNK Cell Immune Response

In addition to activating receptors, a number of inhibitory receptors are differentially regulated in lrNK and cNK cells in these scRNA-seq datasets (Figure 1 and Supplementary Data). The lrNK cell clusters up-regulate genes including *KLRB1, KLRC1*, and *TIGIT*. Conversely, the cNK cell clusters up-regulate *CLEC2D, KIR2DL3, KIR3DL2*, and *KLRG1*.

TIGIT is an inhibitory receptor expressed by T and NK cells and up-regulated upon activation (78, 79). As mentioned previously TIGIT shares its ligands PVR and Nectin-2 with the activating receptor DNAM-I that is up-regulated in cNK cells (80). TIGIT binds with high affinity to PVR and with lower affinity to Nectin-2, with both interactions resulting in inhibition of NK cell cytotoxicity and IFN-γ production (80–82). Hepatocytes and KCs in the liver express PVR equipping them with the ability to directly inhibit NK cell function (83). Hepatocyte PVR levels are increased during liver regeneration, which is suggested to be a mechanism by which hepatocytes safeguard themselves from TIGIT^+^ NK cell killing (83, 84). DCs express both PVR and Nectin-2, and engagement of PVR with a TIGIT-Fc fusion protein increased DC IL-10 production, which may further suppress NK cell function in the liver (79). Using ligand-receptor pair databases Zhang and colleagues predicted that LAMP3^+^ DCs in the liver interact with conventional circulating NK cells via the activating Nectin2-DNAM-I axis and with lrNK cells via inhibitory Nectin-2-TIGIT interactions suggesting that liver DCs may be regulating different NK subsets in opposing directions (85, 86).

KLRD1 (CD94) is up-regulated in both cNK and lrNK cells and can combine with KLRC1 (NKG2A) which is specifically up-regulated in lrNK cells. This heterodimeric complex is a C-type lectin receptor and interacts with the non-classical HLA class I molecule HLA-E on target cells resulting in inhibition of NK cell function. High protein levels of NKG2A have been confirmed on CD49e^−^ intrahepatic NK cells (29, 35), and NKG2A can be induced by the immunosuppressive cytokines TGF-β and IL-10 which are present at high levels in the liver (87, 88). HLA-E is expressed in the liver by both KCs and hepatocytes providing another molecular pathway through which hepatocytes, and KCs, can inhibit lrNK cell activation. In circulating PB NKG2A^+^ NK cells that lack KIR have been shown to kill autologous immature DCs that have reduced HLA-E expression *in vitro* (89), and it is possible lrNK cells are likewise capable of effectively targeting immature DCs that express low levels of HLA-E.

An up-regulation of the *KLRB1* gene in lrNK cells provides a further mechanism that may lead to inhibition of lrNK cell function. Engagement of KLRB1 (CD161) by its ligand lectin-like transcript 1 (LLT1 also known as CLEC2D) inhibits NK cell cytotoxicity and cytokine production (90, 91). LLT1 is expressed by B cells and monocytes and the level of LLT1 decreases on monocytes upon activation (92) but it also increased in cNK cell clusters in these scRNA-seq datasets suggesting that cNK cells may regulate lrNK cell function. While hepatocytes in healthy livers express relatively low levels of LLT1, it is increased in the context of liver cirrhosis and this results in reduced NK cell function (93).

In contrast to lrNK cells, cNK cells express a distinct profile of inhibitory receptors. The scRNA-seq datasets reveal an up-regulation of inhibitory KIRs in cNK cells (*KIR3DL2, KIR2DL3*). This low expression or absence of KIRs on lrNK cells has been validated at the protein level (33). *KLRG1* is also up-regulated in cNK cells. *KLRG1* encodes an inhibitory C-type lectin receptor that recognizes E-cadherin and N-cadherin and inhibits NK cell activation (94).

These differentially expressed inhibitory receptor genes highlight a number of key regulatory mechanisms by which liver hepatocytes and intrahepatic KCs restrain lrNK cell activity and maintain overall organ homeostasis (Figure 2).

### Cytotoxic Machinery

Upon activation by a target cell, NK cells can release cytotoxic granules and up-regulate death-inducing ligands, each of which result in target cell apoptosis. These scRNA-seq datasets indicate an up-regulation of *GZMK* and *TNFSF10* in lrNK cells, and an up-regulation of *GNLY, GZMB*, and *GZMH* in cNK cells (Figure 1 and Supplementary Data).

*TNFSF10* encodes tumor necrosis factor-related apoptosis-inducing ligand (TRAIL), a transmembrane protein that mediates a death-receptor mediated pathway of cytotoxicity. It has been described as important for both NK cell anti-viral and anti-tumor activity in the liver as it binds to TRAIL death receptors (DR) including DR4 and DR5 on target cells to induce target cell-apoptosis (95–97). Decoy receptors (DcR) also exist that do not induce apoptosis and include DcR1 and DcR2. DR4 and DR5 are expressed in by hepatocytes (98), monocytes, monocyte derived macrophages and activated T cells (99).

Granule-mediated cytotoxicity involves the targeted release of secretory granules containing granzymes (e.g., granzyme-A, -B, -H, -K, and -M), together with perforin and granulysin (100). LrNK cells have elevated transcription of *GZMK* which encodes granzyme-K. Granzyme-K has trypsin-like activity and can induce a rapid cell death independently of caspases (100). In contrast cNK cells have elevated expression of *GZMB* encoding for granzyme-B and *GZMH* encoding for granzyme-H. Granzyme-B mediates apoptosis of target cells using caspase-dependent mechanisms (101–103), while granzyme-H can induce cell death independently of caspases (104). Granulysin is a saposin-like protein that has pore forming abilities as well as anti-tumor and anti-microbe cytotoxic activity and is selectively up-regulated in cNK cells (105–107).

The elevated expression of *GZMK* and *TNFSF10* indicates lrNK cells can utilize both death-receptor and granule-mediated cytotoxic pathways to kill target cells, however, they use distinct mediators in comparison to cNK cells where granzyme-B and granulysin are key cytotoxic mediators.

### Regulation of Parenchymal and Non-Parenchymal Cells in the Liver

In addition to mediating cytotoxic responses, NK cells are also capable of producing a wide variety of immunoregulatory molecules, most notably IFN-γ. Within the scRNA-seq datasets lrNK cells upregulate a variety of immunoregulatory genes including *XCL1, XCL2, CCL3*, and *AREG* which may regulate both parenchymal and non-parenchymal cells in the liver and shape the hepatic immune response (Figure 2).

A number of chemokines were identified in lrNK clusters highlighting potential roles in regulating the migration of other immune cell subsets in the liver. XCL1 and XCL2 bind to the XC chemokine receptor 1 (XCR1), which is selectively expressed by cross-presenting CD141^+^ DC's in circulating PB, commonly referred to as cDC1 (108). These cDC1s are functionally equivalent to CD8^+^ DC in the mouse, a subset of DC that endocytose stressed cells and dying cells (109, 110) and preferentially present this processed antigen to CD8^+^ T cells (111, 112). This XCL1-XCR1 axis may provide lrNK cells the ability to regulate DCs within the human liver (38, 39, 113, 114).

The transcripts for *CCL3*, up-regulated in lrNK cells, and *CCL4*, up-regulated in both cNK and lrNK cells, encode CCL3 (also known as MIP-1α) and CCL4 (also known as MIP-1β). Both of these chemokines signal via the CCR1 and CCR5 receptors (115) which are expressed by several cell types including monocytes, NK cells and activated CD4^+^ and CD8^+^ T cells (116, 117). Expression of CCL3 and CCL4 allows lrNK cells to directly regulate migration of myeloid cell populations and T cells into the liver. HSCs also express CCR5 and CCL3-engagement induced their proliferation *in vitro* and implicating the CCL3-CCR5 axis in the progression of liver fibrosis (118, 119).

The liver has a remarkable ability to regenerate itself via hepatocyte proliferation. One of the key pathways involved in regulating hepatocyte proliferation is the epidermal growth factor (EGF) receptor (EGFR). A number of EGFR ligands are known, one of which is amphiregulin (AREG) (120–122), which is up-regulated at a transcriptional level in lrNK cells. Interestingly, *AREG* was only identified in lrNK cell cluster from the Ramachandran et al. dataset. This cluster included lrNK cells from both healthy and cirrhotic liver tissues, suggesting this gene may be up-regulated in lrNK cells in the context of liver disease. AREG is a membrane-bound precursor protein that can be processed and secreted to act in a paracrine or autocrine fashion and is involved in in multiple processes including proliferation, apoptosis and migration of both epithelial and immune cells (123). EGFR is expressed by hepatocytes and HSCs in the liver and the AREG-EGFR axis is known to specifically modulate the hepatic acute phase reaction in the liver regeneration process and is essential for normal hepatocellular survival and proliferation (124, 125). Overall, immune cell derived AREG is suggested to be associated with type 2 immune-mediated resistance and tissue protective mechanisms (123, 126, 127).

## Conclusion

The up-regulation of specific signaling molecules and receptors on lrNK cells and their enrichment in the liver suggest an important liver-specific role for these cells. Attempts to understand these NK cell functions have been limited by the use of functional assays that assess a limited range of markers. Here we show that scRNA-seq datasets can provide novel insights into possible functional roles of human lrNK cells (Figure 2) that may support the development of novel hypotheses to be explored in future functional investigations, once expression at the protein level is validated.

The increased expression of cytotoxic machinery components, including perforin and granzyme-K, equip these lrNK cells with the ability to mediate cytotoxic responses. The activation of this cytotoxicity is tightly controlled by the careful balance of activating and inhibitory receptors, of which lrNK cells express a distinct repertoire. This distinct receptor repertoire may confer the ability to regulate a number of infiltrating immune cell populations within the liver. At the same time this distinct receptor repertoire ensures hepatocytes and KCs can restrain lrNK cell activity and promote organ homeostasis.

Detailed investigations into specific intrahepatic cells that express these receptor ligands and the conditions under which they are induced will aid in our understanding of the liver-specific role of lrNK cells. Validation of potential liver-specific functions of lrNK cells requires a comprehensive analysis of human liver parenchymal and non-parenchymal cell populations, together with the development of novel functional assays. It is evident that a wide diversity of resident intrahepatic NK cell populations exist in the human liver and a detailed understanding of their roles in liver homeostasis and liver disease is essential for our understanding of tissue-resident immune responses within the liver.

## Author Contributions

GJ and MR contributed equally to the conception and design of the article, interpreting the relevant literature, and drafting and reviewing the manuscript. Both authors contributed to the article and approved the submitted version.

## Conflict of Interest

The authors declare that the research was conducted in the absence of any commercial or financial relationships that could be construed as a potential conflict of interest.
